# Improving eye care quality through brief verbal intervention on optometry service provider by using unannounced standardized patient with refractive error: study protocol for a randomized controlled trial

**DOI:** 10.1186/s12886-023-03023-y

**Published:** 2023-06-16

**Authors:** Huijuan Liang, Jiaqi Li, Nan Zhang, Fang Wu, Xiaoshan Chen, Huanyuan Luo, Wenjun He, Siyuan Liu, Ting Kang, Ruotong Zhang, Yujie Liu, Zizhen Huang, Lanping Zhang, Qing Zhao, Sensen Lv, Chunping Li, Yunyun Xie, Dong (Roman) Xu

**Affiliations:** 1 School of Health Management, Inner Mongolia Medical University, Hohhot, China; 2 School of Public Health, Southern Medical University, Guangzhou, China; 3 Acacia Lab for Implementation Science, School of Health Management, Southern Medical University, Guangzhou, China; 4 Acacia Lab for Implementation Science, School of Health Management and Dermatology Hospital, Southern Medical University, Guangzhou, China; 5 Center for World Health Organization Studies, Department of Health Management, School of Health Management of Southern Medical University, Guangzhou, China; 6 Southern Medical University Institute for Global Health (SIGHT), Dermatology Hospital of Southern Medical University (SMU), Guangzhou, China; 7 Acacia Labs, School of Public Health, Southern Medical University, Guangzhou, China

**Keywords:** Refractive error, Optometry service, Eye care, Quality improvement, Brief verbal intervention (BVI), Randomized controlled trial (RCT), Unannounced standardized patient (USP), Parallel-group design

## Abstract

**Background:**

Improper refractive correction can be harmful to eye health, aggravating the burden of vision impairment. During most optometry clinical consultations, practitioner-patient interactions play a key role. Maybe it is feasible for patients themselves to do something to get high-quality optometry. But the present empirical research on the quality improvement of eye care needs to be strengthened. The study aims to test the effect of the brief verbal intervention (BVI) through patients on the quality of optometry service.

**Methods:**

This study will take unannounced standardized patient (USP) with refractive error as the core research tool, both in measurement and intervention. The USP case and the checklist will be developed through a standard protocol and assessed for validity and reliability before its full use. USP will be trained to provide standardized responses during optical visits and receive baseline refraction by the skilled study optometrist who will be recruited within each site. A multi-arm parallel-group randomized trial will be used, with one common control and three intervention groups. The study will be performed in four cities, Guangzhou and three cities in Inner Mongolia, China. A total of 480 optometry service providers (OSPs) will be stratified and randomly selected and divided into four groups. The common control group will receive USP usual visits (without intervention), and three intervention groups will separately receive USP visits with three kinds of BVI on the patient side. A detailed outcome evaluation will include the optometry accuracy, optometry process, patient satisfaction, cost information and service time. Descriptive analysis will be performed for the survey results, and the difference in outcomes between interventions and control providers will be compared and statistically tested using generalized linear models (GLMs).

**Discussion:**

This research will help policymakers understand the current situation and influencing factors of refractive error care quality, and then implement precise policies; at the same time, explore short and easy interventions for patients to improve the quality of optometry service.

**Trial registration:**

Chinese Clinical Trial Registry ChiCTR2200062819. Registered on August 19, 2022.

**Supplementary Information:**

The online version contains supplementary material available at 10.1186/s12886-023-03023-y.

## Introduction

### Background

In 2019, an estimated 2.2 billion people worldwide were vision impaired, and at least 1 billion of them could have been prevented or cured [1]. Myopia is the predominant type of refractive error, and uncorrected refractive error is the leading cause of avoidable visual impairment worldwide [1, 2]. The incidence of myopia is increasing rapidly in China, which also has one of the largest numbers of patients with refractive error [2, 3]. Wearing spectacles provides a cost-effective way to correct refractive error, and optometry is an essential part of refractive correction [4]. However, they are prescribed or dispensed by a wide range of eye care providers, including primary eye care clinicians, ophthalmic personnel, optometrists, ophthalmologists, and personnel without any formal training or professional certification [5]. In addition, the optometry service and functions carried out by each eye care provider are largely dependent on the demand for services and financial incentives in different kinds of eye care facilities, potentially compromising the quality of eye care [6].

Global health faces enormous challenges in providing high-quality eye care in low and middle-income countries [1, 7, 8]. Uncorrected or improperly corrected refractive errors greatly affect patients’ health and quality of life, and even bring a huge economic burden to society [9]. Despite the potential negative effects of poor eye care, few researchers have conducted research on this issue in China. A few studies have discovered a high rate of inaccuracy of spectacle prescriptions. Nearly half (48.8%) of children in rural Guangdong with glasses had an absolute difference in prescription by ≥ 1.00D and 17.7% by ≥ 2.00D [10]. Among children who received optometry in rural western China, 9.1% had poor vision due to inappropriate refractive correction [11]. This study also suggested that rural optometrists were overly dependent on computerized optometry and could not effectively improve vision with subjective optometry. A survey in Shanghai showed that 26.05% of children from migrant families wore glasses with incorrect lenses [12].

Previous studies have shown the prevalence of blindness and visual impairment, and the proportion due to uncorrected or improperly corrected refractive errors. Because of the potential observation bias and recall bias, these studies may provide limited information on the quality of local optometry services. We need a methodology and criteria for establishing quality indicators. In recent years, the unannounced standardized patient (USP) has been increasingly used for healthcare quality measurement in developing countries [13–15]. The USP methodology as the gold standard for quality assessment [16, 17] in clinical practice has the following advantages of being: (a) an objective and direct assessment of care in a real visit; (b) unannounced and thus having no Hawthorne effect (also referred to as the observer effect, in that individuals modify an aspect of their behaviors in response to their awareness of being observed [18]; c) standardized in case presentation and thus providing a natural control for case mix; and d) immediate in terms of recording assessment results and thus having less recall bias. Furthermore, the use of USPs enables us to conduct a randomized controlled trial with multi-arms by creating an experiment condition of the USP case while holding other USP factors constant.

USP encounters were validated as an effective way of measuring reproducibility refractive error within optometry [19]. Sean et al. [6] conducted a cross-sectional survey in Shaanxi, which was the first study to use USPs to assess the quality of eye care in China. Their findings indicated an inaccuracy rate of 25.6% (≥ 1.00D absolute difference) in optometry prescriptions, which was higher than the 18.4% reported in a previous study that did not use standardized patient methods in rural western China [11]. They also found that public hospitals provided poorer quality optometry services compared with private optical shops. Compared with hospitals that generally prescribe rather than dispense spectacles, private optical shops mainly profit from spectacle sales and thus may be incentivized to provide better optometry service to reduce after-sales services for patients with uncomfortable spectacles.

But this USP study still offers areas for improvement. The study used vector dioptric distance (VDD) alone as an outcome indicator for quality. However, VDD has not been validated as a proxy for patients’ refractive tolerance [20], thus, VDD should be used in conjunction with a measure for vision comfort [5, 21]. Otherwise, we need another more reliable indicator, such as optimally prescribed spectacles (or optimal spectacle prescription), for measuring optometry quality (more details in the section on method).

However, those cross-sectional studies only describe the current situation, without continuing to explore the quality improvement of eye care. Along with the promotion of the Healthy China Initiative, eye health policy in the new era has gradually shifted towards high-quality development of eye care. But the change in the policy system is a complex and difficult process, requiring multiple inputs and reforms in various aspects. While the national policy system is undergoing continuous reform, it is beneficial to explore how patients themselves can shape the quality of care during patient-practitioner interactions. Brief verbal intervention (BVI) is an easy and effective method commonly used in primary health services, such as smoking cessation interventions and reducing alcohol intake [22]. In this study, we will use USP to test the effectiveness of BVI in improving eye care quality, especially for optometry services. By utilizing the USP, we are able to vary a specific component (such as a different line used as a form of BVI) across providers while keeping other USP elements constant. As a result, any variations in measurements among the providers can be solely attributed to this particular component.

### Objectives

In summary, the objectives of the study are: (1) to assess the quality of the structure, process, and outcomes of optometry service; (2) to analyze the factors that influence the quality of optometry service; and (3) to assess the effectiveness of patient-based BVI on improving the quality of optometry service.

## Methods

### Study design and setting

The study is designed as a prospective, single-blind, multi-center, randomized controlled trial with four arms – three intervention groups versus a common control group of the participating optometry service providers (OSPs).

The study will be carried out in Guangzhou and Inner Mongolia, China. Guangzhou is the capital city of Guangdong Province, representing a well-developed metropolitan in the south of China. Inner Mongolia’s autonomous region has fewer medical resources, representing relatively underdeveloped land in China. We chose three cities in Inner Mongolia, which are Bayannur (west), Hohhot (central) and Xinganmeng (east). Optometry service providers (OSPs) are considered for inclusion if they meet the criteria defined below. In each location, we will draw a representative sample of eligible OSPs using stratified and simple random sampling.

### Participants and recruitment

**Fig. 1:**
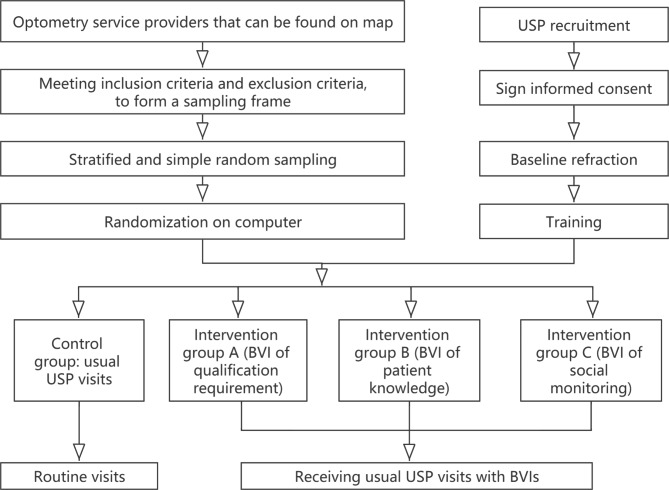
Flow chart of the study

OSPs are not informed of participation, thus they will be visited unannounced by USP. We will recruit USPs and train them, implementing interventions in different groups. Figure 1 presents an explicit flow chart of this study.

The study subjects (OSPs and their refraction-ists) must meet the following criteria to be eligible for the study: (1) OSPs in Guangzhou and Inner Mongolia, consisting of general hospitals with ophthalmology department, ophthalmology specialty hospitals or clinics, and optical shops; (2) any personnel providing optometry services, as licensed optometrists and ophthalmologists or not, at the above-mentioned institutions. Exclusion criteria include: (1) providers providing Internet-based services only; (2) providers providing optometry service solely for contact lenses only.

The USPs must meet the following criteria to be eligible for the study: (1) people with refractive error profile of interest (myopia or myopia with astigmatism), and good ocular health; (2) fluent native speakers of the primary language of the district; (3) having a good memory. If the USPs meet any of the following criteria, they will not be eligible for the study: (1) having prior refractive eye surgery or had eye surgery within the last three months; (2) having manifest or intermittent strabismus, or amblyopia; (3) people with any ocular or health conditions that can cause variable spectacle prescription.

We will develop the sampling frame of all OSPs who meet our inclusion and exclusion criteria by crawling the data of Gaode Map, the leading map service App with the advantage of capturing both registered and non-registered optical shops in China. We will select the sample of OSPs with stratified and simple random sampling. Likewise, we will recruit USPs by posting part-time job announcements on campus posters and social media, like WeChat, and also through our existing USP network. The USPs will be paid for participation and reimbursed for other related expenses.

At recruitment, all USPs and their companions must sign the informed consent electronically through a web link sent by our emails. We have obtained a waiver for informed consent from the OSPs as the process may compromise the sample representation due to possible participant self-selection. We meet the criteria of consent waiver for the USP studies as stipulated by the medial ethics: (1) data analysis will only be performed at the aggregated level rather than targeting individual participants; (2) de-identification for the participating individuals and institutions will be fully implemented; and (3) services performed on USPs are not critical services where resources must be protected for the real patients [23].

### Sample size calculation

We calculated the sample size based on the proportion of optometry accuracy, which is the primary outcome of this study. According to the previous literature [5], the proportion of optometry accuracy is 44.1% in the control group (receiving usual USP visits), and proportions in the intervention groups (receiving usual USP visits plus BVIs) are supposed to be 66.1% (when assuming the effect size of 0.22). Accounting for multiple comparisons of proportions (the proportion of optometry accuracy) for treatments vs. a control [24], the overall alpha is set to be 0.05, thus a level of significance is 0.0167 (0.05/3) by using Bonferroni adjustments. In this study, we set the power of each test at 0.9, all groups have the same sample size. And finally, a total of 456 USP visits is required, each OSP is corresponded to a USP visit. Considering 5% of dropout and/or missing data, the number of participants adds up to a total of 480 participants. The sample size required for the control group and each of the intervention group is 120 respectively. The sample size calculation was performed using PASS 15 software (NCSS, Kaysville, UT, USA).

### Allocation and blinding

The sample of OSPs will be randomly assigned to the control or the intervention groups with a 1:1:1:1 allocation. The randomization schedule will be computer-generated and stratified by service type (such as hospitals and optical shops). A stratified randomization is used to ensure that the four groups have a similar distribution in type as this characteristic may substantially affect the outcomes.

The allocation of OSPs will be conducted by a statistician otherwise not related to the research. Other than a code for each service provider and the service type, the statistician will have no other identifying information of the sampled participants.

After stratified random sampling, the allocation sequence will be generated by computer, and the randomization for each group will be initiated by the researcher. The USPs will be enrolled by the researcher and assigned randomly to the OSPs in each group. To balance confounding factor of individual USP, each USP will be assigned to four groups for equal visits.

The study subjects (OSPs) will be blinded to their allocation status as we have obtained a waiver for their informed consent. The USPs will not be blinded as they will need to implement BVIs according to the allocation status. The outcome assessors and data analysts will be blinded to reduce subjective analysis.

As no informed consent will be sought, unblinding on the side of OSPs is highly unlikely if possible at all. But if that occurs, a replacement of provider with similar characteristics will be identified for a replacement visit.

### Control group

The control group of optical services will receive usual USP visits, while the intervention groups will receive usual USP visits plus a form of BVIs. In the usual USP visit, the customer requests to get their eyes checked for the spectacle prescription, but will otherwise make no other proactive lines. Although a schedule will be developed for each USP to visit sampled OSPs, most visits will be drop-in visits with no prior appointment unless it is a required procedure by the OSP, for example, when USPs have to register online in advance to visit the ophthalmology department of the hospital. The usual USP visit is chosen as the control, because the purpose is to investigate whether patient’s BVI will change practitioner’s behavior and thereby affect the quality of optometry service. It should also be noted that the assessment results in the control group will serve as the cross-sectional survey results of the quality of optometry service.

### Brief verbal interventions

The USP will execute three different forms of BVIs. For each, the USP will deliver exactly the same rendition of the USP cases other than a proactive line before undergoing the eye examination that represents the content of an assigned BVI. The three BVIs are informed by three respective underlying theories: qualification requirement, patient knowledge and social monitoring. In Group A, the USPs will say “I would like to ask for a qualified professional to do the examination/optometry.” or something like that, to create a qualification requirement for better service. In Group B, the USPs will say “I learned from the Internet that subjective refraction is a critical component” to show patients’ knowledge of appropriate procedures. In Group C, the USPs will say “I am a blogger and will share my experience on the Internet” to create a scenario of social monitoring. Except for the first opening statement and BVI, the USPs will not offer any other lines unless prompted by a provider’s question or instruction. They will also provide only standard answers and responses, strictly according to the USP scripts.

BVIs are brief interventions that will be started and concluded with a single USP visit, so we expect few or no cases of discontinuing an allocated intervention. In the case a provider refuses to provide optometry service, the USP will record the reason and visit a replacement provider with similar characteristics.

The intervention will be delivered by highly trained USPs even though they are also real patients. Actually, USP and companion will be trained together, and then always work together. They will be paired according to their free time. The intervention in the study is instant and involves no follow-ups. We will ensure that all participants receive adequate training to ensure the smooth execution of the study. Meanwhile, we will check the quality of USPs’ renditions of their roles and scripts through the voice recordings that the USPs will take with their smartphones during each optical visit. Thus, we expect a high level of adherence to the research protocols, and we will provide feedback to the USPs within 48 h if they do not adhere to the protocols, in order to improve their adherence in further visits.

### Baseline refraction

Refraction of each USP will be conducted prior to the launch of the field visits by the skilled optometrist who will be recruited within each setting to conduct individual refraction. For example, the baseline refraction in Guangzhou will be conducted by experienced optometrists from Zhongshan Ophthalmic Center at Sun Yat-Sen University, the state key laboratory of ophthalmology. Each item of the expert prescription will be used as the “optimal spectacle prescription” (Table 1) for a given USP. The tolerance limits (Table 1) are developed from published studies [5, 25–27] and evaluated through experts’ consensus.

**Table 1:** Criteria for optimal spectacle prescription

Component of spectacle prescription	Tolerance limits compared with baseline prescription
Spherical power	± 0.50DS
Cylindrical power	± 0.50DC
Cylindrical axis	
(if baseline cylinder power ≤ − 0.50DC) (if baseline >−0.50DC to ≤ − 1.50DC) (if baseline >−1.50DC)	± 7° ±5° ± 2°
Pupil distance	± 2 mm
Corrected visual acuity	± 0.1

DS = diopter sphere, DC = diopter cylinder

### Outcomes

The primary outcome is optometry accuracy, a dichotomous variable of spectacle prescription. The prescription of a specific USP visit will be considered “accurate” if all of its readings fall within the tolerance limits in Table 1 to the optimal spectacle prescription. Otherwise, they will be judged as inaccurate prescriptions.

We will also assess providers’ adherence to best practices in optometry services as the secondary outcomes, such as the percentage of guideline-recommended procedures performed. We developed the following technical process indicators based on the guideline recommendations [28]: whether practitioners perform subjective optometry, try on adjustments, check old glasses, etc., and whether the operation is standardized and completed. A technical quality assessment checklist will be developed, and the USP will complete this checklist right after each optical visit. In addition to those technical processes of optometry, we will also evaluate patient experience, cost and service time. The patient-centered eye care in optometry or ophthalmology will be assessed with a revised patient perspective patient-centeredness (PPPC-R) rating scale by USP [29, 30]. Table 2 summarizes all outcomes to be collected.

**Table 2:** Outcomes and their definitions

Name	Definition	Nature	How it is assessed	By whom
optometry accuracy	whether it is optimally prescribed for spectacle	primary outcome	spectacle prescription	USP and companion
completion of process items	the percentage of recommended procedures performed	secondary outcome	quality checklist	USP and companion
patient experience score	whether it is patient-centered	secondary outcome	PPPC-R rating scale	USP
cost	optometry fees, prices of recommended lenses and frames	secondary outcome	data collection form	USP and companion
service time	optometry time and waiting time	secondary outcome	data collection form	USP and companion

### Data collection and management

We will collect a variety of eye care quality information and other related explanatory variables. Donabedian’s classification of quality of care (structure, process and outcome) will be used for quality evaluation [31]. The quality of structure level includes: personnel qualification (whether the practitioner is qualified and professionally certified) and equipment layout (whether the facility’s hardware meet the requirements of optometry and whether its layout is appropriate), etc. This kind of information will be obtained from publicly available websites, promotional materials and USP on-site observations.

Almost all data will be collected through the USP visit to the OSPs. The USP will visit each provider along with a companion as “friend”. While the USP is under examination by the practitioner, the companion will observe the equipment environment and the optometry procedures. At the end of each visit, the USP will ask for a spectacle prescription. The USP and the companion will work together to complete the quality checklist and other data forms on the smartphone within 15 min of the end of the optometry service. We assure that all collected data will be securely stored and protected in accordance with relevant privacy regulations and guidelines.

All data collected will be entered into the Research Electronic Data Capture (REDCap) system by USP companions. REDCap is a free and powerful cloud-based tool for data collection, storage and management [32]. It will also track any changes to the data in an audit trail and be able to fully de-identify the data. REDCap has bank-level security features, and only the investigators involved in this study can access the data. All forms collected for this study, including the signed consent forms, will be stored on REDCap system safely and conveniently.

### Statistical analysis plan

Data will be analyzed on an intention-to-treat (ITT) basis to compare primary and secondary outcomes in the originally assigned groups. Baseline characteristics for each group will be descriptively presented using mean (standard deviation, SD) or median (interquartile range, IQR) for continuous variables depending on the distribution, and frequency (percent) for categorical variables.

Primary outcome (optometry accuracy) and secondary outcomes will be analysed using generalized linear models (GLMs). Binomial distribution and identity link function will be used for binary outcomes, Poisson distribution and identity link function will be used for count outcomes, and normal distribution and identity link function will be used for continuous outcomes. Effects (risk differences for binary and count outcomes, and mean differences for continuous outcomes) with corresponding two-sided 95% confidence intervals will be estimated. Missing data will be imputed using multiple imputation in the GLMs.

Sensitivity analysis will be performed by covariate-adjusted analysis using GLMs with baseline characteristics as covariates. Cost-effectiveness analyses will be performed, and cost-effectiveness ratios will be calculated using bootstrapping techniques. Statistical significance will be considered to be present when the P value is < 0.05. Subgroup analyses will be performed, based on the type of service (hospitals and optical shops) and types of practitioners (such as licensed vs. unlicensed practitioners). IBM SPSS (version 20) and SAS (version 9.4, SAS Institute) will be used for all statistical analyses.

Subgroup analyses will be performed, based on type of service (hospitals and optical shops) and types of practitioners (such as licensed vs. unlicensed practitioners).

We expect the number of withdrawals to be low because the intervention is delivered by the USP and the service provider will receive the intervention passively. Additionally, the intervention will be conducted over a brief period of one USP visit thus without post-visit follow-ups. All data will be entered into the REDCap system immediately after the visit. Thus, we expect to have minimum (if any) missing data. A maximum dropout and/or missing data of 5% is considered in our sample size calculation. We will analyze the data according to the ITT principle.

### Role of implementation

The study will be coordinated between Southern Medical University and Inner Mongolia Medical University. Day-to-day support for the trial is provided by:

- Principle investigator: lead the study design and supervise the conduct of the trial; revise the protocol and amendments; approve the final protocol.
- Executive Investigator: develop research proposal and protocol; select and recruit USPs; conduct sample randomization and allocation; advise on study design and statistical analyses.
- Data manager: organize data capturing system; safeguard data quality; perform statistical analyses.
- Study coordinator: register trial; train USPs; obtain informed consent; coordinate optical service visits; assess quality of the USP visit.
- Study optometrist: develop “optimal spectacle description”; help train USPs.

The study team meets every two weeks. There is no trial steering committee or stakeholder involvement group.

And monitoring of trial conduct will be performed continuously. As the study does not involve any medications or invasive examinations or treatment, we expect minimum harm to the participants. The USPs and their companions will also be encouraged to report any irregularities or concerns through a feedback system. Thus we do not create an independent data monitoring committee for this study.

Protocol modifications will be communicated to the relevant parties in the form of an amendment. In case amendments concern or affect USP participants in any way, they will be informed about the changes. If needed, additional consent will be requested and registered. Also, on-line trial registries will be updated accordingly.

## Discussion

This randomized controlled trial is designed to investigate the effect of BVI by USP on the quality of optometry service. The outcomes will include optometry accuracy, provider adherence to best practices in conducting optometry, and structural elements of the OSPs. The results of this study will not only provide valuable information on the quality of optometry service and identify a specific opportunity for quality improvement but also provide much-needed empirical evidence to aid policy-makers, education institutions and service providers on how to ensure that optical services provide quality eye care. The findings can be especially helpful for people with refractive error to obtain proper refraction and good service as BVIs can potentially be self-implemented by patients. The use of USPs in this study provides a reliable way of measuring and changing quality of the services.

Although we have waived informed consent for the OSPs, we will take strong measures to protect provider information. No service provider or practitioner identification details will be reported in publications or any other public spaces. There is also a way for USPs to prevent getting harm once their anonymity is exposed. All USPs will be intensively trained to present a letter with phone contact information from the university to explain their research roles in case of possible confrontation and also to avoid potentially harmful examination.

There are several limitations that should be considered. First, we will not be able to afford to buy spectacles due to limited budgets. So the results of the data can only reflect the provided optometry service quality, not the whole refractive error care. Second, we do not consider young children, only use adult USPs (aged 18 or up) in the study as mydriatic optometry is an invasive examination that cannot be ethically implemented in the USP study. Third, our USPs have real profiles of refractive error with various diopters, so different USPs may cause the individual difference. We will minimize the potential bias by assigning each USP to the four groups in addition to the random assignment of OSPs. Training will be provided to ensure each USP plays their roles consistently except BVI across groups.

## Conclusion

This randomized controlled trial will provide insight into the effect of BVI in the optical visit. By use of usual visit without BVI as a control group, we will gain insight into the current situation of the quality of optometry service. We focus on technical quality and patient-centeredness, with the gold standard of practice assessment. And USP is a promising tool to not only assess quality but also implement quality improvement interventions. Our study can provide an important contribution to the understanding of refractive error care and the role of BVI as a quality-improvement method, and also provide methodological practice of USPs to future researchers.

## Electronic supplementary material

Below is the link to the electronic supplementary material.

SPIRIT Checklist

## Data Availability

Data sharing is not applicable to this article as no datasets were generated or analysed during the current study.

## Abbreviations

BVI: brief verbal intervention
USP: unannounced standardized patient
RCT: randomized controlled trial
VDD: vector dioptric distance
OSP: optometry service provider
REDCap: Research Electronic Data Capture
SD: standard deviation
IQR: interquartile range
GLM: generalized linear model
